# Evolution, creativity, intelligence, and madness: “Here Be Dragons”

**DOI:** 10.3389/fpsyg.2014.00784

**Published:** 2014-07-23

**Authors:** Rex E. Jung

**Affiliations:** Department of Neurosurgery, University of New MexicoAlbuquerque, NM, USA

**Keywords:** creativity, intelligence, psychosis, autism, abductive, deductive, default mode

One of the great joys of being a scientist is the hunt for an elusive signal within the noise of data, opinions, biases, and other human foibles associated with the pursuit of knowledge. It is inevitable that this imperfect quest will result in many false starts along the way when looking “through a glass, darkly.” Our imperfect and incomplete knowledge of the world must look like an unpolished mirror, reflecting gibberish, at times. However, it also reflects an underlying signal that bears further scrutiny, in spite of our instinct to discard a flawed image of reality. The pursuit of the neural underpinnings of creative cognition is certainly that “dark glass” we peer into so intently, attempting to grasp, through our meager instruments, some hidden truth. Many thinkers and researchers have found that creativity and madness seem somehow to be intertwined, but the signal is weak, the image blurry, and the propensity toward romantic stereotypes is high. And yet, as scientists, we can only follow the data, trying to make sense of what it tells us. So, rather than entertain the premise outright let me take you on a bit of a journey (which will end back at madness, I promise).

First: What if evolutionary processes selected for two types of reasoning? Cosmides and Tooby hypothesized a “dedicated intelligence” that “refers to the ability of a computational system to solve predefined, target set of problems.” These problems often involve well established rules—like your mundane life, and Raven's Matrices problems, and acquiring a language (Pinker, 1991). The other problems require “improvisational intelligence” referring to “the ability of a computational system to improvise solutions to novel problems” (Cosmides and Tooby, 2002). These problems are more transient and involve contingencies that may or may not persist over time—like figuring out how to get into your car, having locked your keys inside. Philosophers call the former type of problem solving “deductive reasoning”—the observations necessarily result in a conclusion being made based on the evidence. They are rule based, deterministic, and the cause leads naturally to effect. The latter problem solving is called “abductive reasoning”—there are an infinite number of possible solutions to the myriad challenges faced in the world; therefore a theory best explains the observation, given the evidence. This reasoning is probabilistic, involves approximation, and (importantly) guessing. Both methods are adaptive: one for problems that are familiar, the other for problems that have never been encountered before.

Kanazawa (2004) views intelligence (incorrectly), the pinnacle of deductive reasoning, as THE domain-specific adaptation to solving novel problems in the environment. However, it is my contention that intelligence and creativity occupy two extremes of a dichotomy: intelligence supplies a “dedicated reasoning capacity” for problems that possess rule-based, cause-effect relationships. Others have covered well, and provide empirical support for, the “general purpose problem solving” capacity of intelligence and “g” (Kaufman et al., 2011): I am merely saying here that the mechanism is rather “dedicated” to cause-effect relationships—a capacity with broad applicability to deductive reasoning tasks. In contrast, creativity emerged as an adaptive cognitive mechanism for low frequency, “improvisational reasoning,” where solutions to problems are unsighted (Simonton, 2013), and probabilistic approximation could lead to novel solutions. Creative reasoning solves the minority of problems that are unforeseen and yet of high adaptability: “The lightning has struck the tree near the camp and set it on fire. The fire is now spreading to the dry underbrush. What should I do?” (Kanazawa, 2004). In this conceptualization, creativity is an evolved cognitive mechanism to abstract, to synthesize, to solve non-recurrent problems in the environment. Finally, intelligence should be seen as a rather stable evolved mechanism over the last 1.6 million years (i.e., the singular “innovation” being the Acheulean hand ax), while creativity appears to have appeared, in humans at least, in the last ~30,000 years (Gabora and Kaufman, 2010). Intelligence may not be evolutionarily novel, but creativity certainly is.

Perhaps the most parsimonious theory of creative cognition to incorporate evolutionary principles is that of Blind Variation and Selective Retention (BVSR) (Campbell, 1960). Indeed, his theory posits that creativity in humans “represent(s) cumulated inductive achievements, stage by stage expansions of knowledge beyond what could have been *deductively* derived from what had been previously known.” Moreover, this creative process possesses three necessary conditions: “a mechanism for introducing variation, a consistent selection process, and a mechanism for preserving and reproducing the selected variations.” Simonton, more recently, assessed and extended BVSR theory, a half-century after its inception, by addressing the shortfalls of Campbell's imprecise definition of what it means for a variation to be “blind:” creativity and discovery are not blind, rather ideas are blind to the extent that the utilities are initially unknown. In contrast, sighted ideas are guided by prior applicable ideas (a.k.a. acquired expertise) (Simonton, 2011). Simonton argues that the “blind variation” component of the theory does not imply that ideas are randomly generated, stating “as long as the probabilities of any generated responses are decoupled from their utilities, the responses are blind without the necessity of being random” (Simonton, 2011). Campbell's notion of BVSR provides an evolutionary framework for creative cognition and has emerged as a “universal selection theory” for numerous other disciplines ranging from neuroscience, to computer science, to philosophy (Simonton, 2010).

Moreover, in Campbell's framework, creative thought represents a simulation or “substitution” of representations of the environment in ones mind, with the “solution” being selected from the numerous thought experiments undertaken, “according to a criterion which is in itself substituting for an external state of affairs.” When put into action, the selectively retained solution results in “intelligent behavior” (if adaptive) as opposed to blind floundering. Campbell provides numerous examples of thinkers relying upon “chance combinations” of ideas that appear to coalesce into workable solutions, with Poincaré most famously describing five stages of creative thought (later trimmed to four) including preparation, incubation, illumination, and verification (Poincaré, 1908). All have in common the notion of “trial and error” thinking resulting in an “insight” or “solution” that appears to be most adapted to a given problem in the world. An important implication of the BVSR model is that the results of creative thought are rather disconnected from their antecedents—it is not sufficient to have great minds in order to have creative solutions, just many minds and/or many variations: “insofar as there has been a genuine gain in knowledge, the difference between a hit and a miss lies in the selective conditions thus newly encountered, not in talent differences in the generation of the trials.”

Taken one step further, and with Cambell's dichotomy of BVSR in mind, it is not a great leap of imagination to posit that: the “dedicated” cognitive mechanism resides within conscious awareness, with full access to memory stores, planning, attention, and action algorithms serving smooth allocation of resources toward adaptive responses to ongoing, predictable, environmental demands. Measures of such dedicated cognitive mechanisms, termed “IQ” should be (and indeed are) highly correlated with nearly all measures of adaptive ability, including height, health, education, occupation, income, longevity—a staggering array of fitness indicators (Gottfredson, 1997). Stretching our imagination a bit more, we might infer that the improvisational mechanism (unfortunately almost exclusively measures of divergent thinking) will be inconsistently, negatively, and/or weakly correlated with measures of adaptive fitness due to the very low recurrence of such environmental problems (Kanazawa, 2004), the inadequacy of measurement of the underlying cognitive construct (Arden et al., 2010), and the poor correlation between antecedents and their ultimate effective solutions (Simonton, 2014).

In the next step, we can now synthesize the cognitive systems with hypothesized neural mechanisms: the dedicated system is likely to rely upon EXPLICIT or conscious knowledge, while the improvisational system relies more upon IMPLICIT or unconscious knowledge systems (Helie and Sun, 2010). The interaction of explicit and implicit systems can be seen to form the basis of effective, adaptive problem solving within an organism required to solve both common and novel problems in the world. Finally, at a neural network level, the explicit/dedicated system would appear to have significant overlap with the cognitive control network, while the implicit/improvisational system would appear to overlap significantly with the default mode network (Jung et al., 2013).

But what of madness? This is where we really must stretch our thinkers to hypothesize where things might go awry, as they always do, out in the messy world of biological beings. Two competing mechanisms are at play in the human brain, one driving toward abstraction, the other toward certainty. At the far extreme of one end of this highly adaptive bell curve resides psychosis: all things are linked together; all things are related to me; all things are relevant (manifesting as delusions, hallucinations, disorganized speech/behavior). The link between creative genius and psychoticism is not new, having been explored by Eysenck with regard to that rare bird “genius” (Eysenck, 1995). However, true psychosis is a rather rare phenomenon—the lifetime incidence being around 3%, as opposed to “madness” in general (Perälä et al., 2007). At the other extreme is adherence to rigid, rule-based, behavior—the far reaches of which might naturally encompass autism spectrum disorders (ASD's)—also very rare, with a recent total population prevalence found to be 2.64% (Kim et al., 2011). This dichotomy (i.e., psychosis/autism) is not a new hypothesis, having been recently (and brilliantly) applied to “the social brain,” (Crespi and Badcock, 2008). Nor is it a radical departure from Carson's “shared vulnerability model,” (Carson, 2013) although factors leading to extremes of either creativity (e.g., cognitive flexibility, low latent inhibition) or intelligence (e.g., cognitive closure, high sensitivity) are seen to be pathological in the current model. What is new is to apply this dichotomy to the reasoning brain as manifested through intelligent and creative pursuits (Figure 1).

**Figure 1:**
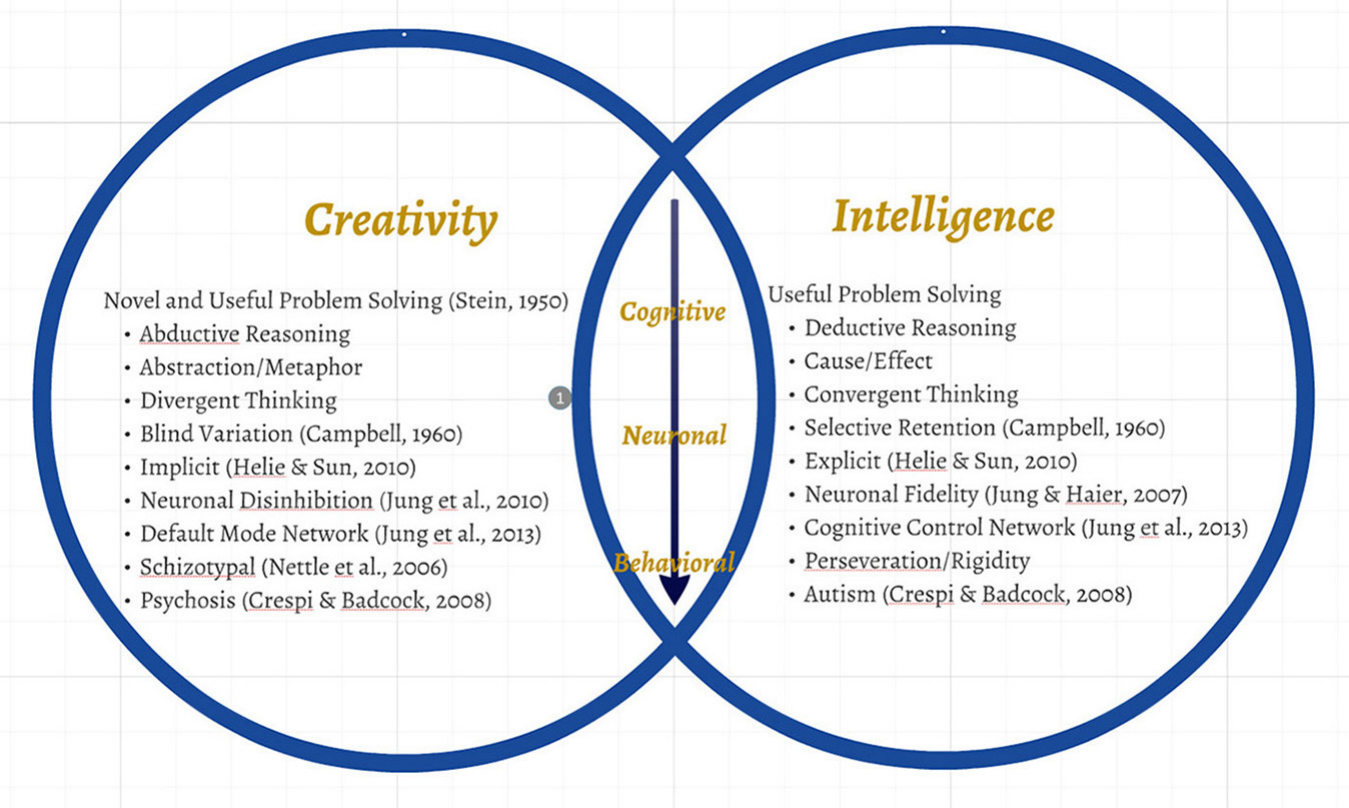
**Proposed model for dichotomy of reasoning pressures on human evolution with cognitive, neuronal, and behavioral correlates**.

Can one be both “mad” (i.e., overtly psychotic) and creative? Certainly no evidence exists that creative genius (or even garden variety creativity) lurks, emerges, or is unleashed in the presence of overt psychosis (or autism for that matter, savants notwithstanding). Might these examples of “madness” reside at the extreme ends of continua that produced more adaptive levels of flexibility and order (a.k.a. novelty vs. usefulness) (Stein, 1953)? Certainly possible, and increasing evidence suggests this to be so (Nettle, 2006; Glazer, 2009; Kyaga et al., 2011; Fink et al., 2012). Are all of these ideas empirically testable? Indeed, they are—and should be—through falsifiable hypotheses as opposed to anecdote, hyperbole, or press release. But beware! When on a scientific journey, looking “through a glass, darkly,” one might see all sorts of strange things at the far edges of the known world—some even breathing fire—but shedding little light (Dietrich, 2014).

## Conflict of interest statement

The authors declare that the research was conducted in the absence of any commercial or financial relationships that could be construed as a potential conflict of interest.

## References

[B1] Arden R. Chavez R. S. Grazioplene R. Jung R. E. (2010). Neuroimaging creativity: a psychometric view. Behav. Brain Res. 214, 143–156 10.1016/j.bbr.2010.05.015 20488210

[B2] Campbell D. T. (1960). Blind variation and selective retention in creative thought as in other knowledge processes. Psychol. Rev. 67, 380–400 10.1037/h0040373 13690223

[B3] Carson S. H. (2013). Creativity and psychopathology: shared neurocognitive vulnerabilities, in Neuroscience of Creativity, eds Vartanian O. Bristol A. S. Kaufman J. C. (Cambridge, MA: The MIT Press), 175–203

[B4] Cosmides L. Tooby J. (2002). Unraveling the enigma of human intelligence: evolutionary psychology and the multimodular mind, in The Evolution of Intelligence, eds Sternberg R. J. Kaufman J. C. (Mahwah, NJ: Erlbaum), 145–198

[B5] Crespi B. Badcock C. (2008). Psychosis and autism as diametrical disorders of the social brain. Behav. Brain Sci. 31, 241–261 10.1017/S0140525X08004214 18578904

[B6] Dietrich A. (2014). The mythconception of the mad genius. Front. Psychol. 5:79 10.3389/fpsyg.2014.00079 24616710 PMC3935122

[B7] Eysenck H. J. (1995). Genius: The Natural History of Creativity. Cambridge: Cambridge University Press 10.1017/CBO9780511752247

[B8] Fink A. Slamar-Halbed M. Unterrainer H. F. Weiss E. M. (2012). Creativity: genius, madness, or a combination of both. Psychol. Aesthetics Creativity Arts 6, 11–18 10.1037/a0024874

[B9] Gabora L. Kaufman S. B. (2010). Evolutionary approaches to creativity, in The Cambridge Handbook of Creativity, eds. Kaufman J. S. Sternberg R. J. (Cambridge, UK: Cambridge University Press), 279–300

[B10] Glazer E. (2009). Rephrasing the madness and creativity debate: what is the nature of the creativity construct? Pers. Individ. Diff. 46, 755–764 10.1016/j.paid.2009.01.021

[B11] Gottfredson L. S. (1997). Mainstream science on intelligence: an editorial with 52 signatories, history, and bibliography (Reprinted from The Wall Street Journal, 1994). Intelligence 24, 13–23 10.1016/S0160-2896(97)90011-8

[B12] Helie S. Sun R. (2010). Incubation, insight, and creative problem solving: aunified theory and a connectionist model. Psychol. Rev. 117, 994–1024 10.1037/a0019532 20658861

[B13] Jung R. E. Mead B. S. Carrasco J. Flores R. A. (2013). The structure of creative cognition in the human brain. Front. Hum. Neurosci. 7:330 10.3389/fnhum.2013.00330 23847503 PMC3703539

[B14] Kanazawa S. (2004). General intelligence as a domain-specific adaptation. Psychol. Rev. 111, 512–523 10.1037/0033-295X.111.2.512 15065920

[B15] Kaufman S. B. Deyoung C. G. Reis D. L. Gray J. R. (2011). General intelligence predicts reasoning ability even for evolutionarily familiar content. Intelligence 39, 311–322 10.1016/j.intell.2011.05.002

[B16] Kim Y. S. Leventhal B. L. Koh Y. J. Fombonne E. Laska E. Lim E. C. (2011). Prevalence of autism spectrum disorders in a total population sample. Am. J. Psychiatry 168, 904–912 10.1176/appi.ajp.2011.10101532 21558103

[B17] Kyaga S. Lichtenstein P. Boman M. Hultman C. Långström N. Landén M. (2011). Creativity and mental disorder: family study of 300 000 people with severe mental disorder. Br. J. Psychiatry 199, 373–379 10.1192/bjp.bp.110.085316 21653945

[B18] Nettle D. (2006). Schizotypy and mental health amongst poets, visual artists, and mathematicians. J. Res. Pers. 40, 876–890 10.1016/j.jrp.2005.09.004

[B19] Perälä J. Suvisaari J. Saarni S. I. Kuoppasalmi K. Isometsä E. Pirkola S. (2007). Lifetime prevalence of psychotic and bipolar I disorders in a general population. Arch. Gen. Psychiatry 64, 19–28 10.1001/archpsyc.64.1.19 17199051

[B20] Pinker S. (1991). Rules of language. Science 253, 530–535 10.1126/science.1857983 1857983

[B21] Poincaré H. (1908). L'invention mathematique. Bull. Inst. Gen. Psychol. 8, 175–187

[B22] Simonton D. K. (2010). Creative thought as blind-variation and selective-retention: combinatorial models of exceptional creativity. Phys. Life Rev. 7, 156–179 10.1016/j.plrev.2010.02.002 20416854

[B23] Simonton D. K. (2011). Creativity and discovery as blind variation: Campbell's 1960 BVSR model after the half-century mark. Rev. Gen. Psychol. 15, 158 10.1037/a0022912

[B24] Simonton D. K. (2013). Creative thought as blind variation and selective retention: why creativity is inversely related to sightedness. J. Theor. Philos. Psychol. 33, 253–266 10.1037/a0030705

[B25] Simonton D. K. (2014). Can creative productivity be both positively and negatively correlated with psychopathology? Yes! Front. Psychol. 5:445 10.3389/fpsyg.2014.00455 24904471 PMC4033124

[B26] Stein M. I. (1953). Creativity and culture. J. Psychol. 36, 311–322 10.1080/00223980.1953.9712897

